# Nanoengineered polyaniline/carbon black VXC 72 hybridized with woven abaca for superior electromagnetic interference shielding

**DOI:** 10.1038/s41598-025-99521-8

**Published:** 2025-04-25

**Authors:** Martin Guillermo C. Fernandez, Muhammad Luthfi Hakim, Zufar Alfarros, Gil Nonato C. Santos, Muhammad Akhsin Muflikhun

**Affiliations:** 1 Physics Department, De La Salle University, Manila, Philippines; 2 Mechanical and Industrial Engineering Department, Universitas Gadjah Mada, Yogyakarta, Indonesia; 3 Department of Electrical Engineering Education, Universitas Negeri Yogyakarta, Yogyakarta, Indonesia; 4 Center for Energy Studies, Universitas Gadjah Mada, Yogyakarta, Indonesia

**Keywords:** Engineering, Mechanical engineering

## Abstract

The growing demand for efficient electromagnetic (EM) shielding materials has driven extensive research into sustainable and functionalized composites for high-frequency applications. This study investigates the electromagnetic (EM) shielding properties of Polyaniline (PAni)-functionalized woven abaca fibers, reinforced with Carbon Black (CB) VXC 72, in the ultrahigh-frequency (UHF) range (500–4500 MHz), as determined using Vector Network Analyzer (VNA). The composite was developed by functionalizing abaca fabric with PAni through in situ chemical oxidative polymerization and depositing CB via a dip-and-dry method. The morphological structure and elemental composition were analyzed using scanning electron microscopy (SEM) and energy-dispersive X-ray spectroscopy (EDX), while Fourier-transform infrared (FTIR) spectroscopy was used to confirm functional group interactions. Electrical resistivity was determined using the four-point probe method, and EMI shielding effectiveness (SE) was evaluated in the ultrahigh frequency (UHF) range of 500 MHz to 4500 MHz using a Vector Network Analyzer (VNA). Experimental results indicate that PAni/CB functionalization successfully imparted shielding properties to abaca fabric. PAni/1CB/Abaca exhibited the highest shielding effectiveness with an average SE of 5.96 dB, corresponding to 74.34% attenuation of incident electromagnetic waves, and a peak attenuation of 7.45 dB at 4.5 GHz. In contrast, 2CB/Abaca and PAni/2CB/Abaca showed selective EMI shielding, with peak attenuation values of 8.27 dB at 1.67 GHz and 7.98 dB at 1.69 GHz, respectively. The electrical resistivity measurements revealed that PAni/1CB/Abaca had the lowest resistivity at 891 Ω·cm, whereas 1CB/Abaca exhibited the highest at 5238 Ω·cm. The primary shielding mechanism was absorption rather than reflection, making the composite a lightweight, corrosion-resistant alternative to traditional metal-based EMI shields. These findings demonstrate the potential of natural fiber-based conductive composites for flexible EMI shielding applications in telecommunications, healthcare, and aerospace industries.

## Introduction

In today’s rapidly evolving technological landscape, electromagnetic interference (EMI) mitigation is a major concern to ensure the reliability and safety of electronic systems, as well as support a wide range of applications in nanotechnology, medicine, and energy^1–3^. As electronic devices become increasingly integrated into daily life, the demand for effective EMI shielding materials continues to grow, driving innovation in both material science and engineering^4,5^. Electromagnetic Interference (EMI) is interference to electronic components or systems caused by unwanted electronic signals transmitted via radiation or conduction^6^. This interference contributes to the increasing risk of electronic “pollution”, which can negatively impact telecommunications, cybersecurity, and health^7^. Rapid advances in electronics and communications technology have highlighted the need for shielding materials designed to protect devices and individuals from electromagnetic interference (EMI). EMI shielding materials must have sufficient electrical conductivity to effectively interact with electromagnetic waves through a conduction loss mechanism. The effectiveness of this shielding relies heavily on the material’s ability to form a strong conductive network, thus allowing efficient movement of charge carriers and increasing its capacity to attenuate electromagnetic interference^8–10^. The current wearable EMI shielding market is dominated by metal-based materials, such as Nickel-Copper/Nylon, Silver/Nylon, and Steel/Nylon. However, these materials have several limitations, including susceptibility to corrosion, high density, as well as high manufacturing costs due to the scarcity of certain metals^11–14^.

Among the vast family of intrinsically conductive polymers, polyaniline (PAni) has garnered significant attention due to its exceptional electrical properties, lightweight nature, and ease of processing^15,16^. These characteristics make PAni a highly promising material for the development of advanced multifunctional composites, particularly in applications requiring enhanced electrical conductivity, electromagnetic interference (EMI) shielding, and energy storage capabilities. Additionally, PAni exhibits remarkable environmental stability, tunable conductivity through protonation, and compatibility with various reinforcing materials, further expanding its potential for integration into diverse high-performance systems. Its ability to form effective conductive networks, even at relatively low concentrations, makes it an attractive candidate for fabricating lightweight yet highly efficient electronic components, flexible sensors, and smart coatings. As research continues to explore innovative polymer-based materials, the synergistic combination of PAni with other functional fillers, such as carbon-based nanomaterials^17–20^.

Conducting polymers such as polyaniline have been explored as potential lightweight, corrosion-resistant substitutes for metals in applications like Electromagnetic Interference (EMI) shielding^21–23^. The development of a well-established conductive network plays a crucial role in achieving effective electromagnetic interference (EMI) shielding, as it facilitates the efficient dissipation and reflection of electromagnetic waves. Materials must possess inherently high electrical conductivity or be reinforced with a sufficient concentration of functional fillers that enhance their conductive properties to attain optimal shielding performance. These fillers, such as carbon-based materials, metal nanoparticles, or hybrid composites, improve the overall shielding efficiency by increasing charge carrier mobility and optimizing the material’s ability to absorb or reflect electromagnetic radiation^24–26^. The balance between intrinsic conductivity and filler loading is critical, as excessive filler content may compromise mechanical flexibility or processability, while insufficient conductivity may reduce shielding effectiveness.

Similarly, conductive carbon allotropes like Carbon Black VXC 72 have emerged as alternatives due to their excellent mechanical, thermal, and electrical properties^27–29^. Their unique characteristics make them ideal for various applications, including energy storage devices, conductive coatings, sophisticated composites, and electronic components. Carbon Black VXC 72 outstanding electrical conductivity improves its usefulness in batteries and supercapacitors, while its good thermal stability enables it to resist extreme working temperatures. Furthermore, its mechanical strength adds to the durability and lifespan of the materials in which it is used, making it an essential component in high-performance engineering and industrial applications^30,31^. However, some previous studies are still limited in exploring the effectiveness of Carbon Black VXC 72 in hybrid composites for electromagnetic interference shielding applications. Combined, these two materials enhance the conductivity and aspect ratio of the composite, leading to improved shielding performance. Additionally, these materials can be applied to textile surfaces, providing enhanced mechanical stability and facilitating the direct capability of the nanomaterial^32,33^. Bio-inspired approaches such as the use of polynorepinephrine and MXene in the form of magnetic nanohybrids have shown high potential for electromagnetic interference shielding in the X-band range while providing strain-sensing functions^34^. On the other hand, the development of acoustic wave-based green synthesis methods to produce graphene-gallium nanoparticles and PEDOT: PSS hybrid coatings is also an innovative strategy in mitigating electromagnetic radiation pollution in textiles^35^. In line with this trend, advances in smart EMI materials and materials with well-organized multilayer structures further strengthen the effectiveness of electromagnetic shielding^36,37^. In addition, the utilization of renewable resources, such as oxidized cellulose nanocrystals from durian peel waste, as well as the development of nanocrystal-reinforced polyester/flax fiber composites, demonstrate efforts to combine high mechanical performance with environmental sustainability^38,39^.

This research addresses the gap in electromagnetic interference (EMI) shielding materials by exploring the potential of Polyaniline (PAni) and Carbon Black (CB) functionalized on abaca fiber textiles, a novel approach that has not been extensively studied. Previous studies have largely focused on metal-based materials such as nickel-copper and silver for EMI shielding, but these materials suffer from drawbacks like corrosion, weight, and high costs. By combining PAni and CB on a natural fiber substrate like abaca, this study presents a lightweight, flexible, and sustainable alternative. This research offers a novelty in improving the effectiveness of EMI shielding through an absorption mechanism rather than reflection, with PAni-CB-Abaca composites exhibiting superior conductivity and more stable EMI attenuation than previous materials. The EMI shielding properties are influenced by the porous structure of the abaca fiber that strengthens wave reflection and absorption, as well as the interfacial polarization between the filler and fiber, which together support the attenuation of EM energy into heat. This natural fiber-based approach also opens up opportunities for sustainable applications in the fields of smart textiles, portable electronic shielding, as well as electromagnetic protection for military, communication, and healthcare purposes.

## Materials and methods

### Synthesis of polyaniline/abaca (PAni/Abaca)

**Fig. 1:**
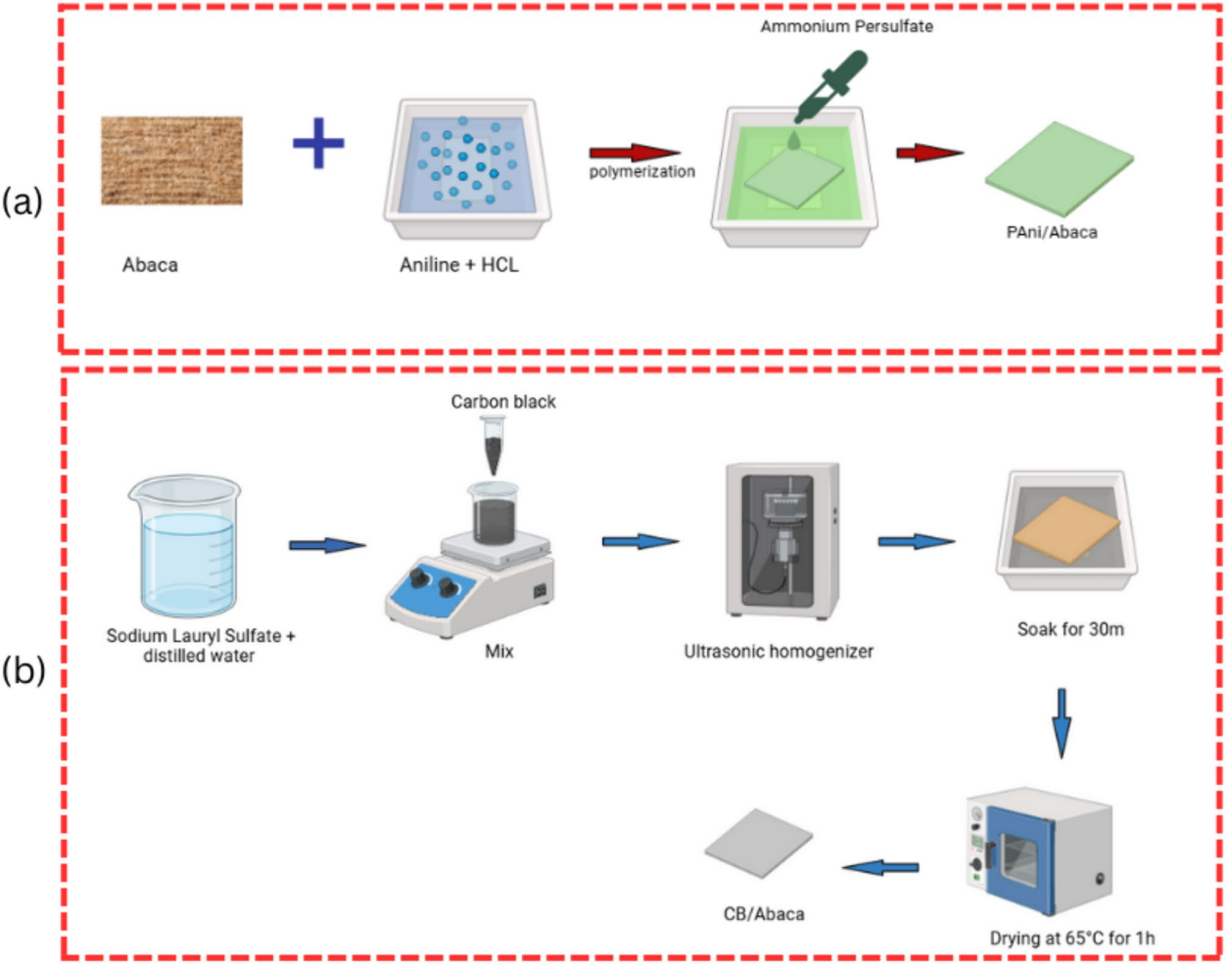
(**a**) Schematic representation of Pani/Abaca fabrics synthesis, (**b**) schematic representation of CB/Abaca coating process.

 Polyaniline/Abaca was synthesized through in situ chemical oxidative polymerization, following:1.25 a monomer-to-oxidant molar ratio^40^, and modified from methods for PAni/textiles proposed in previous^41,42^. The complete stages of this synthesis process can be seen in Fig. 1(a). In this study, the process begins with the immersion of Abaca fibers in a diffusion solution consisting of 100 mL of 1 mL of aniline monomer solution in 0.2 M HCl for 2 h, with the condition of the solution covered with aluminum foil to prevent photodegradation and surrounded by ice to maintain a temperature close to 2 °C so that the polymerization takes place under control. The polymerization process begins with the addition of 100 mL of a solution of 2.85 g of Ammonium Persulfate (APS) in 0.2 M HCl gradually (dropwise) into the diffusion solution, followed by a reaction for 1 h while ensuring the temperature remains low with the addition of ice if necessary. Once polymerization is complete, the PAni/Abaca Composite is collected using antistatic tweezers. It undergoes a repeated washing stage, which involves rinsing with 0.2 M HCl to remove unbound PAni, distilled water to remove excess acid residue, and ethanol to wash away any contaminants that may remain. After washing, the material is air-dried overnight to ensure optimal structural and morphological stability. Thus, this synthesis method produces PAni/Abaca composites with good polymer retention and potentially superior conductive and mechanical properties, making them viable candidates for conductive material-based applications and functional textiles.

### Adhesion of carbon black/abaca and formation of ternary composite

Coating Carbon Black (CB) on Abaca fibers is carried out using the dip-and-dry method, which begins with manufacturing CB-based conductive inks to ensure a homogeneous dispersion. The Dip-and-Dry method is a simple technique used to coat a material with a conductive solution or ink through repeated soaking and drying processes to ensure even adhesion and distribution. The complete stages of this coating process can be seen in Fig. 1(b). A total of 5.4 g of Sodium Lauryl Sulfate (SLS) was dissolved in 180 mL of distilled water (H₂O), then stirred mechanically to remove agglomeration before magnetic stirring at a speed of 475 rpm. After the solution is homogeneous, the specified mass of CB is introduced into the solution and stirred for 15 min, and then this conductive ink solution is ultrasonicated for 5 min to ensure an even distribution of CB particles in the liquid medium. The coating process is carried out by immersing an Abaca fiber cloth in 60 mL of CB ink for 30 min, during which the fabric is turned over with antistatic tweezers mid-immersion to ensure uniform adhesion over the entire fiber surface. After the soaking process is complete, the fabric is dried in the oven at a temperature of 65 °C for 1 h, then allowed to stand for 10 min at room temperature before washing. Washing is carried out by immersion in distilled water to permanently remove Unbound CB and SLS residues, then re-drying in the oven to ensure that no moisture remains. In addition, in the manufacture of tertiary composites (ternary composites), Abaca substrates that have been coated with CB are subsequently polymerized in situ with Polyaniline (PAni), where CB acts as a nucleation point for more stable and evenly distributed PAni growth. This process produces Abaca fiber-based composites with a more conductive and homogeneous material structure, which have the potential to be used in conductive smart materials and textile applications.

### Characterization

 The composites’ surface morphology and elemental composition were analyzed using a PHENOM XL Desktop Scanning Electron Microscope (SEM) and Energy Dispersive X-ray Spectroscopy (EDX) at the iNano Satellite Laboratory. The samples were sputter-coated with gold using a JEOL JFC 1200 Fine Coater to mitigate surface charging and minimize noise. Functional groups present in each composite were identified via Fourier Transform Infrared Spectroscopy (FTIR) using the Nicolet 6700 FTIR Spectrometer at the DLSU Chemistry Instrumentation Room. For FTIR analysis, samples were finely ground with Potassium Bromide (KBr) using a mortar and pestle, formed into pellets, and loaded into the spectrometer after background data was collected and subtracted from the spectra. The electrical resistivity of the composites was evaluated through the Four-Point Probe method, employing a LODESTAR MPS 3005LP-3 as the current source, an ASTI digital multimeter as the ammeter, and an Agilent 34,410 A digital multimeter at UST-RCNAS. Swatches measuring 2 × 1 cm² were prepared, and resistivity measurements were taken across a range of applied voltages (1–5 V).

## Results

### Characteristics of the abaca composites

**Fig. 2:**
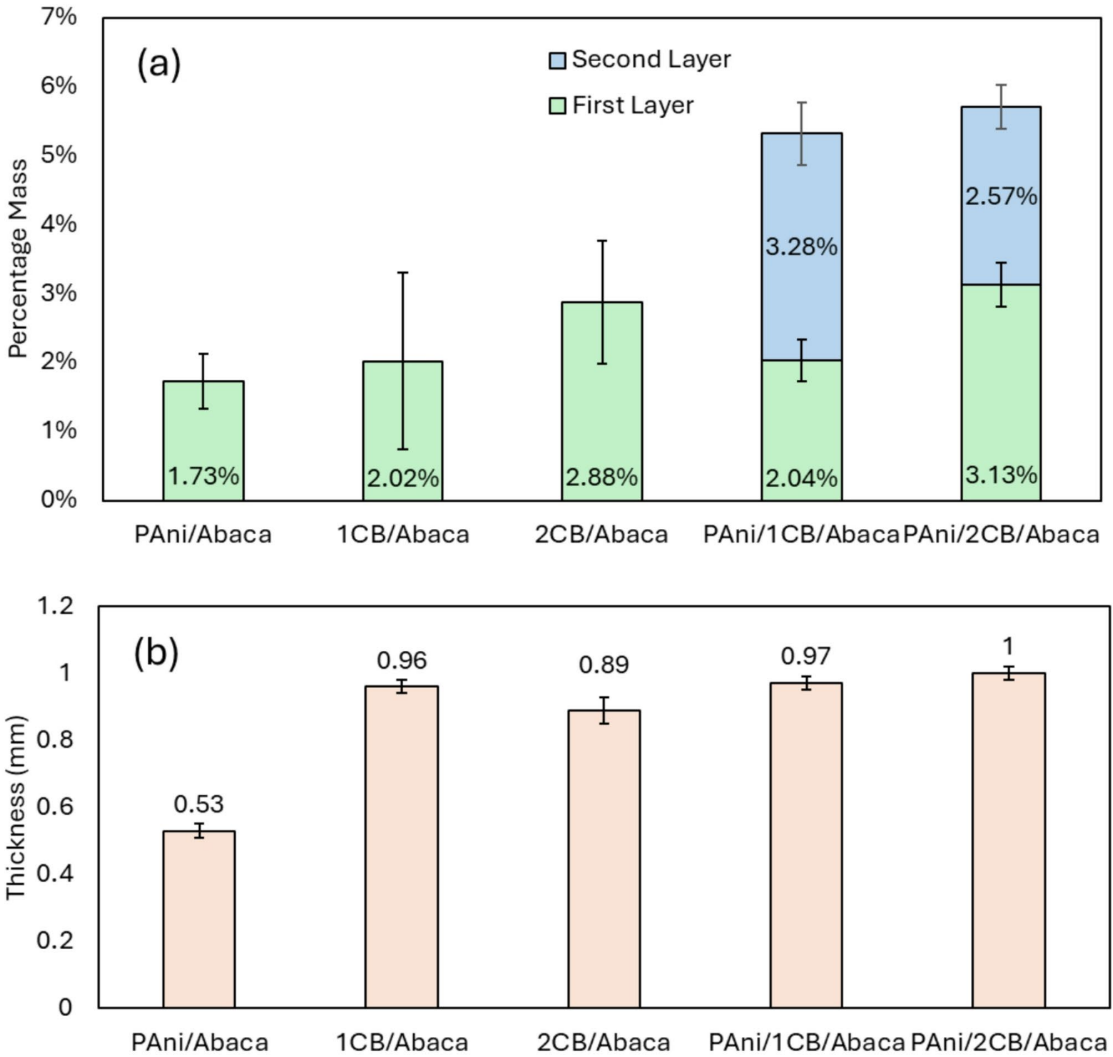
Relative Mass measurements of the specimens. (**a**) Mass percentages, (**b**) Thickness (mm).

The deposition of nanomaterials on Abaca fibers depends on the physical properties of the fibers, which in turn influences the available sites for nanomaterial attachment. Variations in mass are expected due to fiber fraying during processing, but efforts were made to recover these fibers during the weighing process. Figure 2 offers an estimate of the nanomaterial deposition for a 13 × 13 cm^2^ area, showing that the increase in mass due to the nanomaterials is minimal, a vital factor for scalability. Figure 2(a) shows the PAni/1CB/Abaca composite has the highest mass percentage of 3.28%, followed by PAni/2CB/Abaca (2.57%), indicating that the presence of carbon black (CB) acts as a nucleation point for the growth and stabilization of PAni polymer chains. This allows the formation of a longer and interconnected polymer structure, thereby increasing the mobility of electric charges in the composite material^43^. In addition, the role of CB in increasing PAni retention has been confirmed in several studies, showing that CB not only functions as a nucleation point but can also reduce the formation of uneven aggregates in the PAni structure, directly contributing to the improvement of polymer distribution^44^. The difference in nanomaterial deposition between 1CB/Abaca and 2CB/Abaca is likely due to the excessive CB saturation in 2CB/Abaca, which blocks the active area on the fiber for further PAni polymerization, thus limiting the amount of PAni that can be effectively deposited. Figure 2(b) shows the thickness of the composites, where the PAni/2CB/Abaca-based composite has the highest thickness, while the PAni/Abaca composite has the lowest thickness of 1 and 0.53, respectively.

In contrast, 2CB/Abaca (2.88%), 1CB/Abaca (2.02%), and PAni/Abaca (1.73%) showed lower relative masses, indicating that the addition of PAni significantly increased the retention of nanomaterials compared to CB alone. This indicates that PAni has a higher affinity for Abaca fibers, especially under well-controlled in situ polymerization conditions. The physical conditions of the Abaca fibers, including fiber distribution and possible fiber shearing during the process, also influence variations in nanomaterial deposition. However, a recovery step was performed before the final weighing. This non-uniform distribution may cause minor disturbances in the final results, although the relatively small standard deviations (± 0.3%) in PAni/1CB/Abaca and PAni/2CB/Abaca indicate relatively uniform PAni deposition despite the structural variations in the Abaca fibers. Thus, these results suggest that the combination of PAni and CB provides enhanced deposition efficiency. Still, excessive CB proportion may limit PAni exclusion, as seen in PAni/2CB/Abaca, which has a lower PAni mass than PAni/1CB/Abaca. This may be due to CB saturation on the fiber surface blocking the active area required for optimal PAni polymerization. CB saturation creates a physical layer that covers the active groups on the fiber surface, thereby inhibiting the chemical interactions required for effective PAni chain formation during the polymerization process. These implications suggest that optimizing the CB to PAni ratio is critical to producing composite materials with high conductivity and light mass characteristics for large-scale applications. In addition, CB dispersion control strategies may be an important factor in supporting the success of PAni polymerization and avoiding aggregation effects that can reduce material efficiency.

### Surface morphology and energy dispersive X-ray analysis

**Fig. 3:**
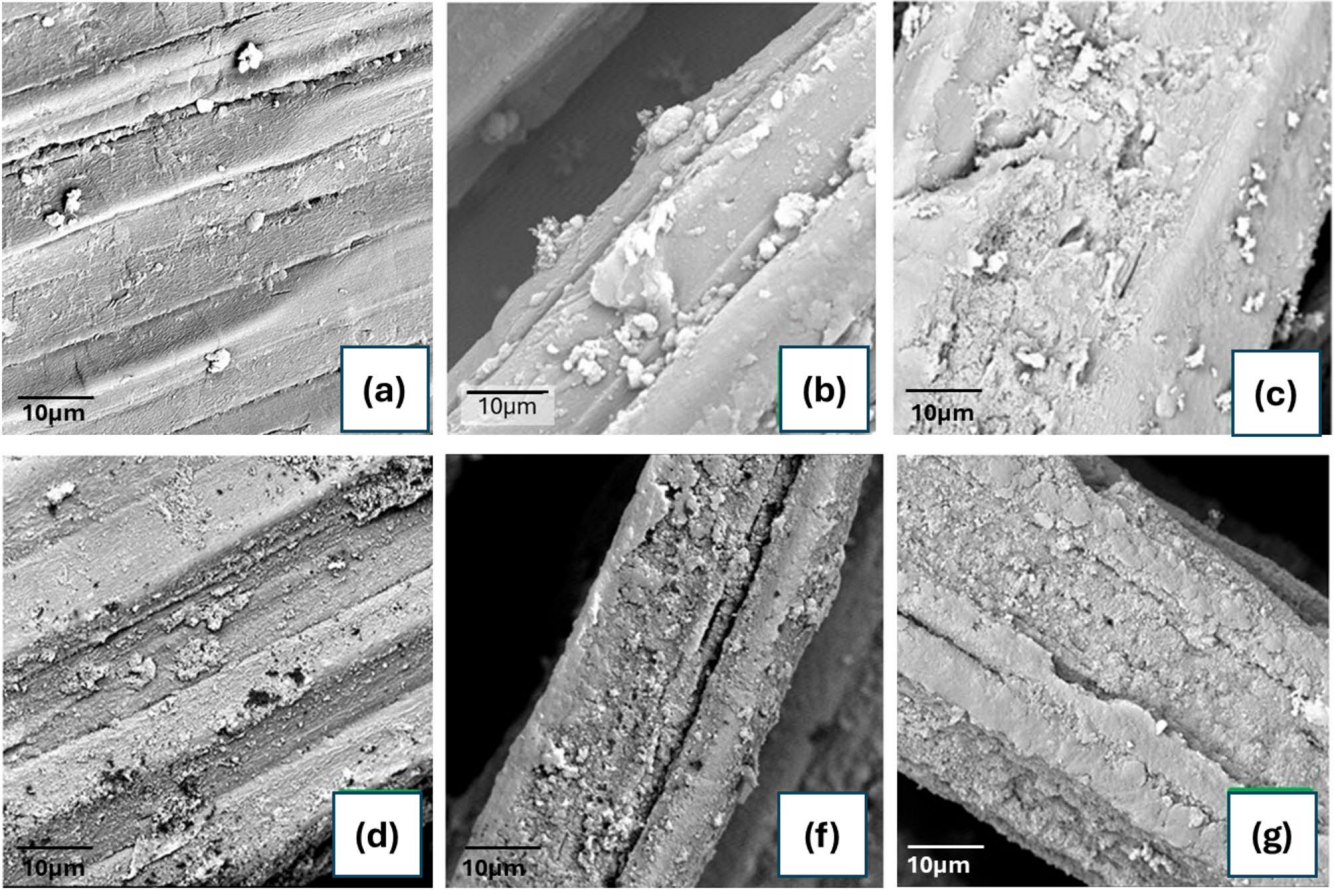
SEM Micrographs of (**a**) Pristine Abaca, (**b**) PAni/Abaca, (**c**) 1CB/Abaca, (**d**) 2CB/Abaca, (**e**) PAni/1CB/Abaca and (**f**) PAni/2CB/Abaca.

Figure 3 shows SEM observations of pristine Abaca, PAni/Abaca, 1CB/Abaca, 2CB/Abaca, PAni/1CB/Abaca and PAni/2CB/Abaca samples. The morphology of pure abaca can be recognized by its coarse fiber assemblage, with flat disc flakes of about 2 μm evenly distributed across the fiber surface as shown in Fig. 3(a). Figure 3(b) shows an increase in the density of PAni particles along the fiber surface, which may indicate the formation of more stable bonds through adsorption or in-situ polymerization mechanisms. This phenomenon indicates that PAni is not only in the form of granular particles distributed on the surface but also begins to completely envelop the fiber. The structure of PAni is in the form of cauliflower-like aggregates, which is consistent with the findings of previous studies^42,45^. Figure 3(c) shows the morphology of 1CB/Abaca, where CB has filled the surface of Abaca. The 2CB/Abaca composite shows a significant increase in aggregation on the surface, characterized by rougher edges as well as greater surface area coverage compared to the 1CB composite (Fig. 3(d)). The addition of Polyaniline to the PAni/1CB/Abaca composite showed better distribution of Polyaniline on the Abaca surface compared to its counterpart using only PAni (Fig. 3(e)). The better distribution of PAni in PAni/1CB/Abaca is due to the presence of an optimal amount of CBs acting as anchor and nucleation points, thus promoting more even adsorption and growth of polyaniline along the fiber surface without causing excessive agglomeration. The significant increase in mass in Abaca compared to its counterpart suggests that the distribution of CB particles in this sample acts as an anchor, allowing PAni to adhere better around the fibers^46,47^. The morphological profile of the PAni/2CB/Abaca rough edges were similar to that of 2CB/Abaca (Fig. 3(f)). The formation of the granular layer indicates the formation of PAni which indicates the correct layer-by-layer deposition on the CB layer. At this mass concentration of CB, the diffusion time may not be sufficient for the polyaniline to completely envelop or fill the CB layer which explains the breaks in the polymer matrix^27^. The prior exposure of the coating may also imply that the coating could be damaged due to the poor mechanical stability of the observed nanomaterials. It can also be argued that the conductive network is damaged during the post-polymerization period. PAni forms a granular layer in PAni/2CB/Abaca because the high concentration of CB causes the formation of large aggregates that interfere with the uniform growth of the PAni layer. In addition, the diffusion time of aniline monomer during the in-situ polymerization process becomes insufficient to form a continuous layer, so PAni tends to precipitate in the form of granular particles.

**Fig. 4:**
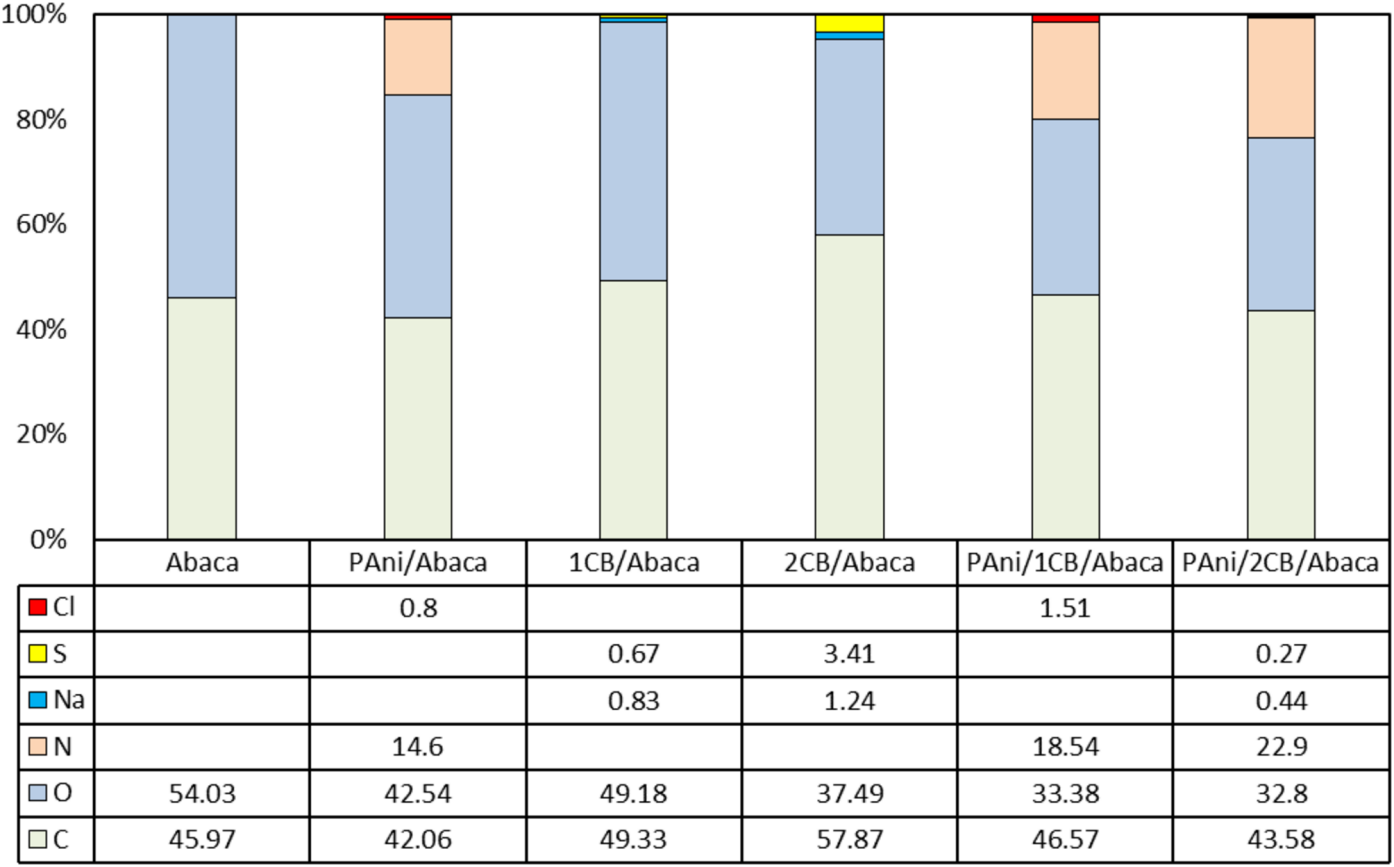
EDX compilation of the Abaca and functionalized composites.

Figure 4 shows the results of elemental composition analysis on Abaca-based composites, showing that Polyaniline deposition (PAni) was successfully performed, as indicated by the high nitrogen (N) content in all samples containing PAni. PAni/2CB/Abaca has the highest nitrogen content (22.9%), followed by PAni/1CB/Abaca (18.54%) and PAni/Abaca (14.6%), which indicates that carbon black (CB) acts as a nucleation point that increases the attachment of PAni to the abaca fiber substrate. Previous studies have shown that CB can accelerate polymer percolation and improve cohesion between layers in conductive composite materials, thus supporting these findings^48^. In addition, CB-containing samples showed low sodium (Na) and sulfur (S) levels, indicating successful surfactant leaching, with the exception of 2CB/Abaca, which had higher Na and S levels of 1.24% and 3.41%, respectively, which likely contributed to agglomeration in the material. In contrast, PAni/2CB/Abaca shows lower Na and S levels, most likely due to additional leaching during synthesis. Nitrogen content correlates with improved PAni attachment because nitrogen atoms are a major structural part of the PAni polymer chain. The higher the nitrogen content detected, the more PAni molecules successfully bound to the Abaca fiber surface, thus reflecting the successful deposition and attachment quality of PAni in the compost.

Significant differences were also found in chlorine content (Cl), where PAni/2CB/Abaca had no detectable Cl content, in contrast to PAni/Abaca (0.8%) and PAni/1CB/Abaca (1.51%), indicating that PAni in PAni/2CB/Abaca may have lower levels of conductivity due to loss of CL doping effects. PAni doped with Cl usually has higher conductivity, so the absence of Cl in PAni/2CB/Abaca may indicate reduced conductive properties. Reduced Cl can occur due to more aggressive leaching processes or interactions with CB that inhibit PAni optimal doping, which requires further optimization. In addition, the percentage of oxygen (O) in PAni-based samples decreased compared to pure Abaca, indicating that PAni and CB replaced some of the oxygen groups present in Abaca fibers. The decrease in oxygen levels in PAni-based samples compared to pure Abaca indicates the substitution of oxygen groups with PAni and CB, which is in line with the in situ polymerization process. Meanwhile, carbon (C) has the highest proportion in 2CB/Abaca (57.87%), reflecting the predominance of carbon black in such composites, which can affect the material’s mechanical properties and pore structure. The higher carbon content in 2CB/Abaca affects the mechanical properties of the composite because the high number of carbon black particles can cause agglomeration, form weak points in the composite structure, reduce cohesion between the matrix and fibers, and increase the brittleness of the material. This makes the material less elastic and more prone to cracking or mechanical failure^49^. Overall, these results suggest that the combination of PAni and CB plays a role in increasing polymer retention on Abaca substrates, but the elemental composition in PAni/2CB/Abaca shows indications of decreased conductivity, leading to the need for further optimization in material formulation in order to achieve optimal conductive properties.

### Fourier transform infrared (FTIR) peak analysis

**Fig. 5:**
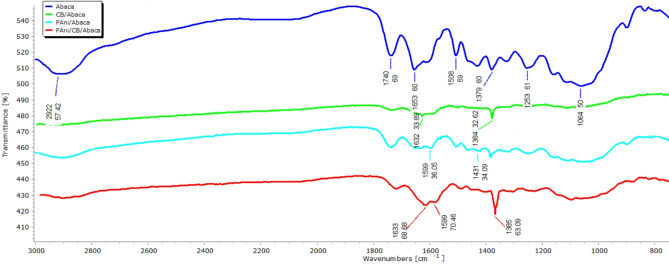
FTIR Transmittance Data of the Abaca composites.

 Figure 5 shows the FTIR spectra of various samples, confirming the presence of structural overlap between Polyaniline (PAni) and natural components in Abaca fibers. This is evidenced by the various absorbance peaks identified in the spectrum. A typical functional group of PAni was detected at a peak of 1599 cm⁻1, indicating the presence of a Quinoid ring [N = Q = N], as well as at a peak of 1431 cm⁻1, indicating a Benzenoid ring [N-B-N]^15,50^. Meanwhile, the presence of Carbon Black (CB) is more difficult to identify directly because its molecular structure has a limited dipole. This leads to a decrease in transmittance intensity in the FTIR spectrum, so the characterization of CB requires a more specific approach. Nonetheless, indications of the presence of CB can be traced through the appearance of a weak peak at 1632 cm⁻1, leading to a carboxyl group (-COO), as well as an increase in peak intensity of 1384 cm⁻1, indicating the presence of -OH bending, as described by Tsubokawa^51^. Thus, these results confirm that PAni is successfully deposited on Abaca fibers, while CB affects material properties by decreasing transmittance, demonstrating its modification potential in these composites.

### Resistivity and theoretical values of SE

**Fig. 6:**
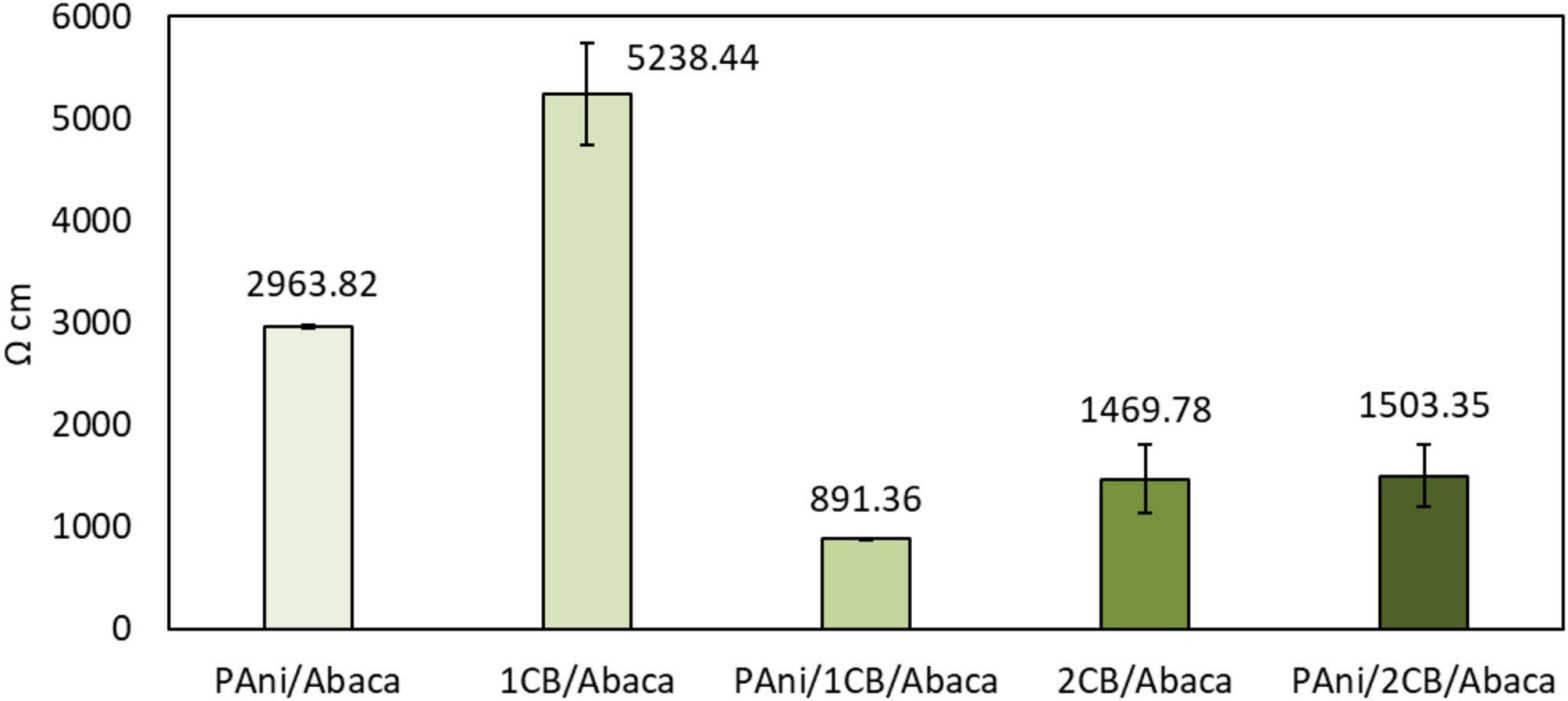
Electrical resistivity values of the Abaca composites.

Figure 6 shows the results of abaca-based composite electrical resistivity measurements are strongly influenced by the type and amount of conductive material used. The 1CB/Abaca composite has the highest resistivity value (5238 ± 499 Ω cm), which can be attributed to the random dispersion pattern of carbon black (CB), which causes the formation of a non-optimal conduction path. The uneven morphological structure inhibits connectivity between particles, reducing the effectiveness of electron transport and increasing the variability of measurements seen from considerable standard deviations. In contrast, the PAni/1CB/Abaca composite showed the lowest resistivity value (891 ± 3 Ω cm) with a low standard deviation, indicating a more homogeneous distribution of the nanomaterial and the formation of a more efficient conductive path. PAni conductive chain formation increases the mobility of charge carriers, which is superior to the CB self-organizing mechanism in providing electrical percolation pathways. PAni enhances charge carrier mobility more effectively than CB alone because PAni forms long conjugated polymer chains that allow delocalization of electrons along the polymer backbone, thus accelerating charge movement. In contrast, CB relies solely on inter-particle contact which requires very close inter-particle spacing to form a conduction pathway, which is prone to resistance at the inter-particle boundary. In the samples 2CB/Abaca (1469 ± 78 Ω·cm) and PAni/2CB/Abaca (1503 ± 15 Ω·cm), there was an increase in conductivity compared to 1CB/Abaca, which can be attributed to better surface gap filling and improved connectivity of the CB matrix, consistent with the findings reported by Hakim et al.^18^ However, the presence of PAni in PAni/2CB/Abaca does not seem to have reached optimal electrical percolation, likely due to an imbalance between the amount of CB and the polymerization conditions of PAni. This finding confirms that in addition to the amount of CB, polymerization time and PAni distribution largely determine the efficiency of the conduction pathways in these composites. Therefore, the optimization of PAni deposition parameters as well as the proportion of CB are key factors in the development of Abaca fiber-based composite materials for conductive applications.

### Shielding effectiveness

The EMI-SE measurements indicate that the absorption (SE_A_) mechanism dominates all Abaca composites, with the reflective portion (SE_R_) negligible. This is consistent with expectations, as materials with significant SE_R_ values (≥ 1 dB), typically metals, are known for having a high number of free electrons^52^. Additionally, the amorphous structure of carbon black (CB) contributes to the low reflection of electromagnetic waves. The relative magnetic permeability (µ_r_) of non-magnetic materials is approximately equal to 1, as demonstrated by prior studies and confirmed through experimental research^52–54^. The SE_A_ evaluation of the composites show that PAni/1CB/Abaca attenuates 5.96 dB on average and a maximum of 7.45 dB at 4.5 GHz, showing a significant increase from its components (PAni/Abaca SE_A_: average = 1.12 dB; max = 3.00 dB @ 540 MHz and 1CB/Abaca SE_A_: average = 1.47 dB; max = 3.86 dB @ 4.32 GHz). As for 2CB/Abaca and PAni/2CB/Abaca, the composites performed almost similarly with a SE_A_ of 3.27 dB and 2.64 dB, respectively. In the context of electromagnetic (EM) shielding absorption, power is directly proportional to the square of the electric field intensity. Shielding effectiveness is defined as the logarithmic ratio between the incident and transmitted power^6^. Additionally, based on Eq. 1, for an arbitrary attenuated electric field (E) at a depth (z), approaching a specific thickness (d), the ratio of the initial electric field intensity (E₀) to E follows this Eq. 1.

1 $$\:{SE}_{A}\left(dB\right)=20\text{log}\frac{1}{{e}^{-\alpha\:z}}=20\:\text{l}\text{o}\text{g}{\:e}^{\alpha\:d}$$

2 $$\:S{E}_{A}\left(dB\right)=20\alpha\:z\text{log}e=8.68\:\alpha\:d$$

Electromagnetic Shielding Effectiveness (EMI-SE) is quantified as a logarithmic function representing the ratio of incident power (P_i_) to transmitted power (P_t_)^6^. P_i_ is composed of three key components: reflected power (P_r_), absorbed power (P_a_), and transmitted power (P_t_). These power components are typically expressed as fractions of P_i_, forming the Reflection Coefficient (R), Absorption Coefficient (A), and Transmission Coefficient (T), respectively^55^. Furthermore, the coefficients R and A are commonly referred to as “Reflection Loss” and “Absorption Loss”, respectively.

3 $$\:{SE}_{Total}\left(dB\right)\equiv\:10\text{log}\frac{{P}_{i}}{{P}_{t}}$$

4 $$\:{P}_{i}={P}_{r}+\:{P}_{a}+{P}_{t}$$

5 $$\:1=R+A+T$$

In experiments, the Scattering Parameters (S_mn_) detected by the two-port Vector Network Analyzer (VNA) can be used to determine the coefficients R, A, and T of a sample under test (SUT), where “m” denotes the port receiving the signal and “n” denotes the port transmitting it^45,55^. It is possible to determine A by changing the values of R and T. According to ASTM 4935 requirements, the SUT is positioned between two coaxial flanged cables, at which point a transverse electromagnetic wave strikes the sample. The detectors read the S-parameters, and the VNA interprets them in real-time. The scattering parameter measurement scheme used was adopted from previous research conducted by Maestre and Santos^2^.

6 $$\:R=\:{\left|{S}_{11}\right|}^{2}or\:{\left|{S}_{22}\right|}^{2}$$

7 $$\:T=\:{\left|{S}_{12}\right|}^{2}or\:{\left|{S}_{21}\right|}^{2}$$

The SUT’s total shielding effectiveness (SE_T_) can be decomposed into its reflected and absorbed components.

8 $$\:{SE}_{T}\left(dB\right)=10\text{log}\frac{1}{T}$$

The effectiveness of electromagnetic interference shielding (EMI-SE) can also be represented as a percentage (SE%) by comparing the signal attenuation relative to a reference signal, as suggested by Bonaldi, et al.^53^. Based on Eq. 9, it can be inferred that an SE value of 20 dB corresponds to a shielding efficiency of 99.0% against incoming electromagnetic waves, making the material suitable for commercial applications^55^.

9 $$\:SE\%=100\times\:\left(1-\left(\frac{1}{{10}^{SE/10}}\right)\right)\:\:$$

**Fig. 7:**
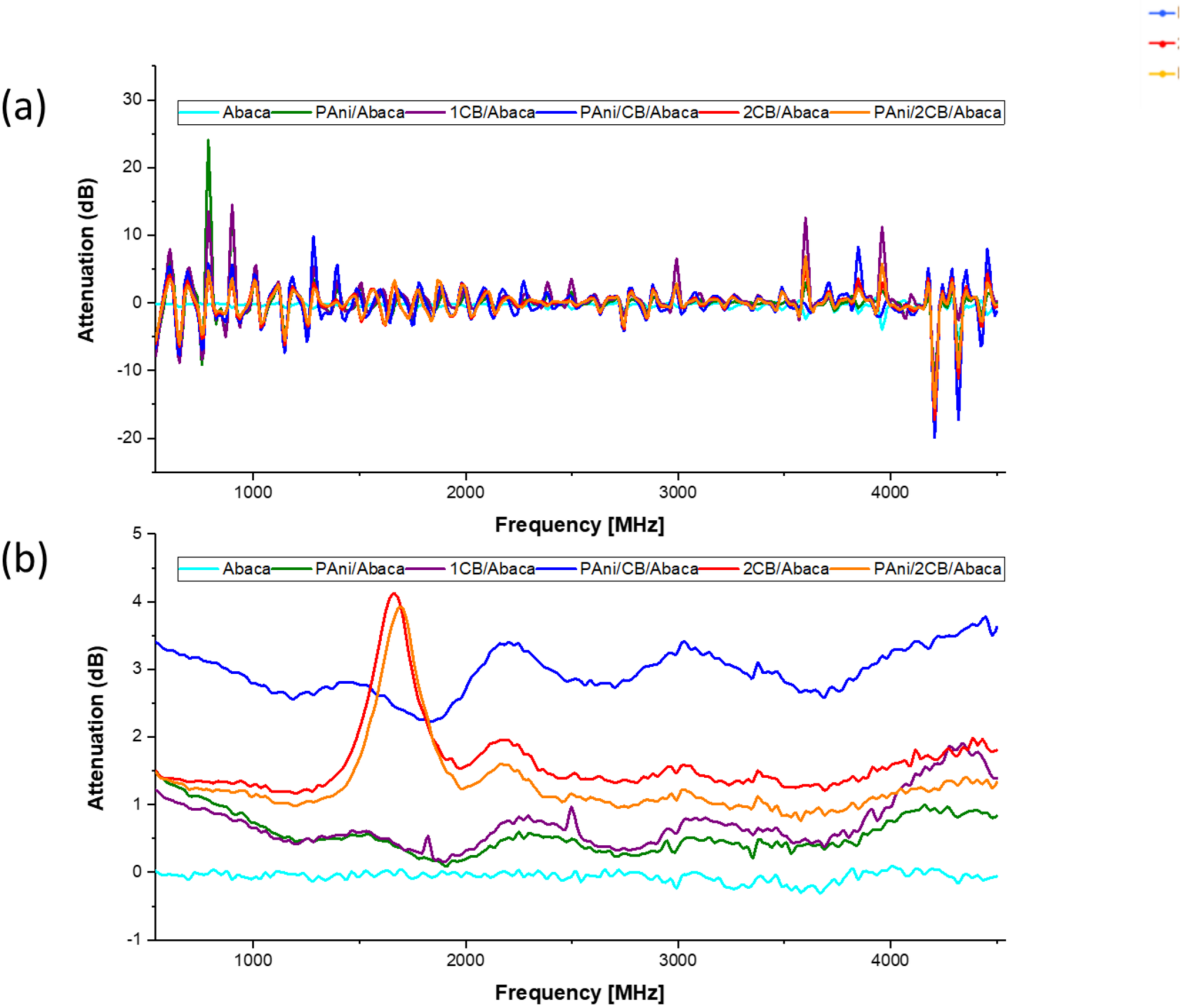
Logarithmic S-parameters (**a**) S_11_ and (**b**) S_21_.

**Fig. 8:**
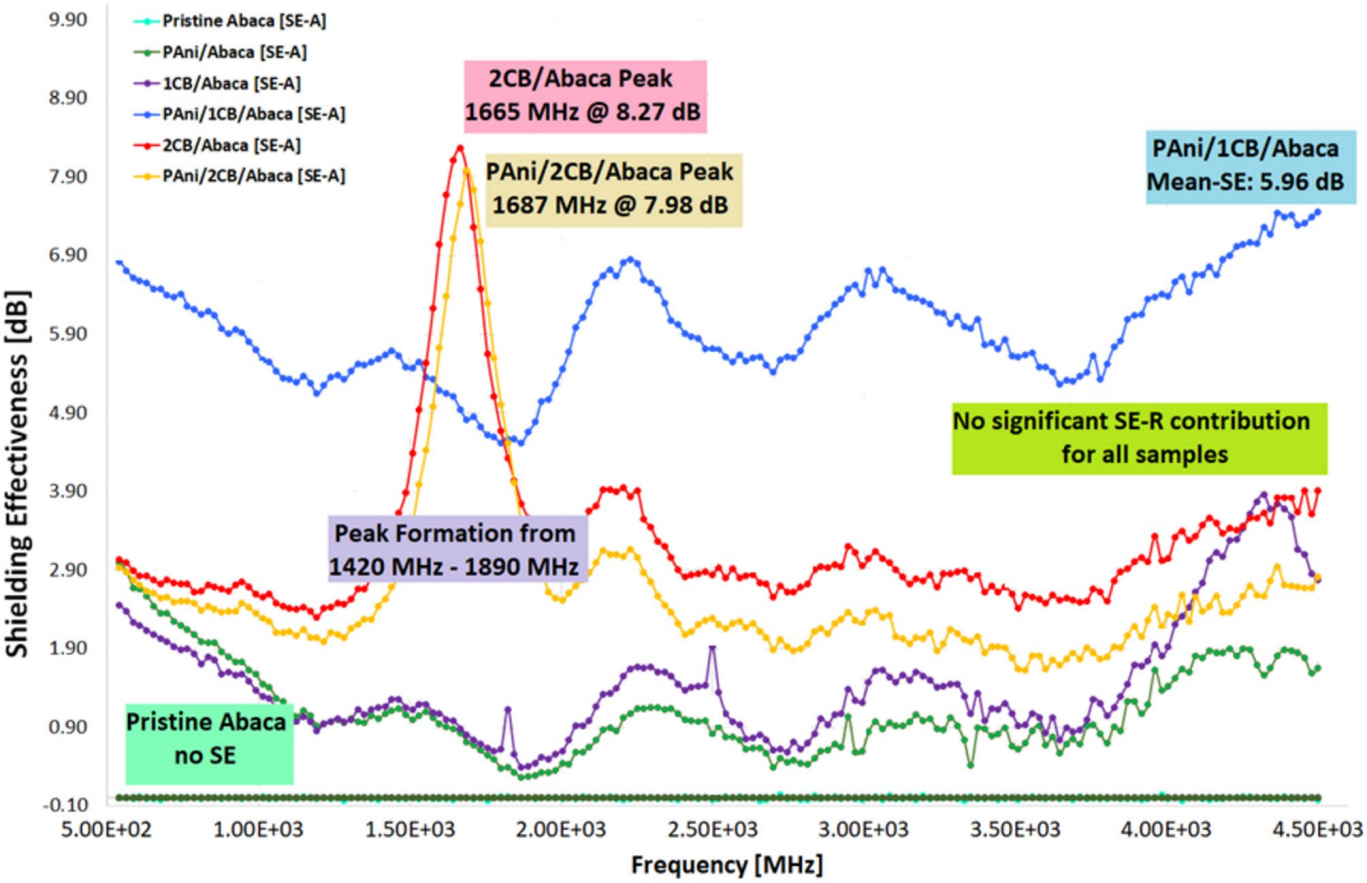
Electromagnetic shielding effectiveness of the abaca composites.

The measured S_11_ and S_21_ parameters of the whole sample can be seen in Fig. 7. Figure 7(a) shows the S_11_ parameter which represents the signal reflection; the attenuation value is low to -20 dB, especially in PAni/2CB/Abaca, indicating good impedance matching. Meanwhile, Fig. 7(b) illustrates the S_21_ parameter, with the PAni/CB/Abaca and 2CB/Abaca combinations showing the highest signal absorption around 2000 MHz, indicating their potential as EMI shielding materials. Figure 8 presents the SE_A_ analysis, showing that the PAni/1CB/Abaca composite achieves an average attenuation of 5.96 dB, peaking at 7.45 dB at 4.5 GHz, significantly outperforming its individual components. Specifically, PAni/Abaca shows an average SEA of 1.12 dB with a maximum of 3.00 dB at 540 MHz, while 1CB/Abaca has an average SE_A_ of 1.47 dB and a maximum of 3.86 dB at 4.32 GHz. The 2CB/Abaca and PAni/2CB/Abaca composites exhibit similar performance, with SE_A_ values of 3.27 dB and 2.64 dB, respectively.

**Fig. 9:**
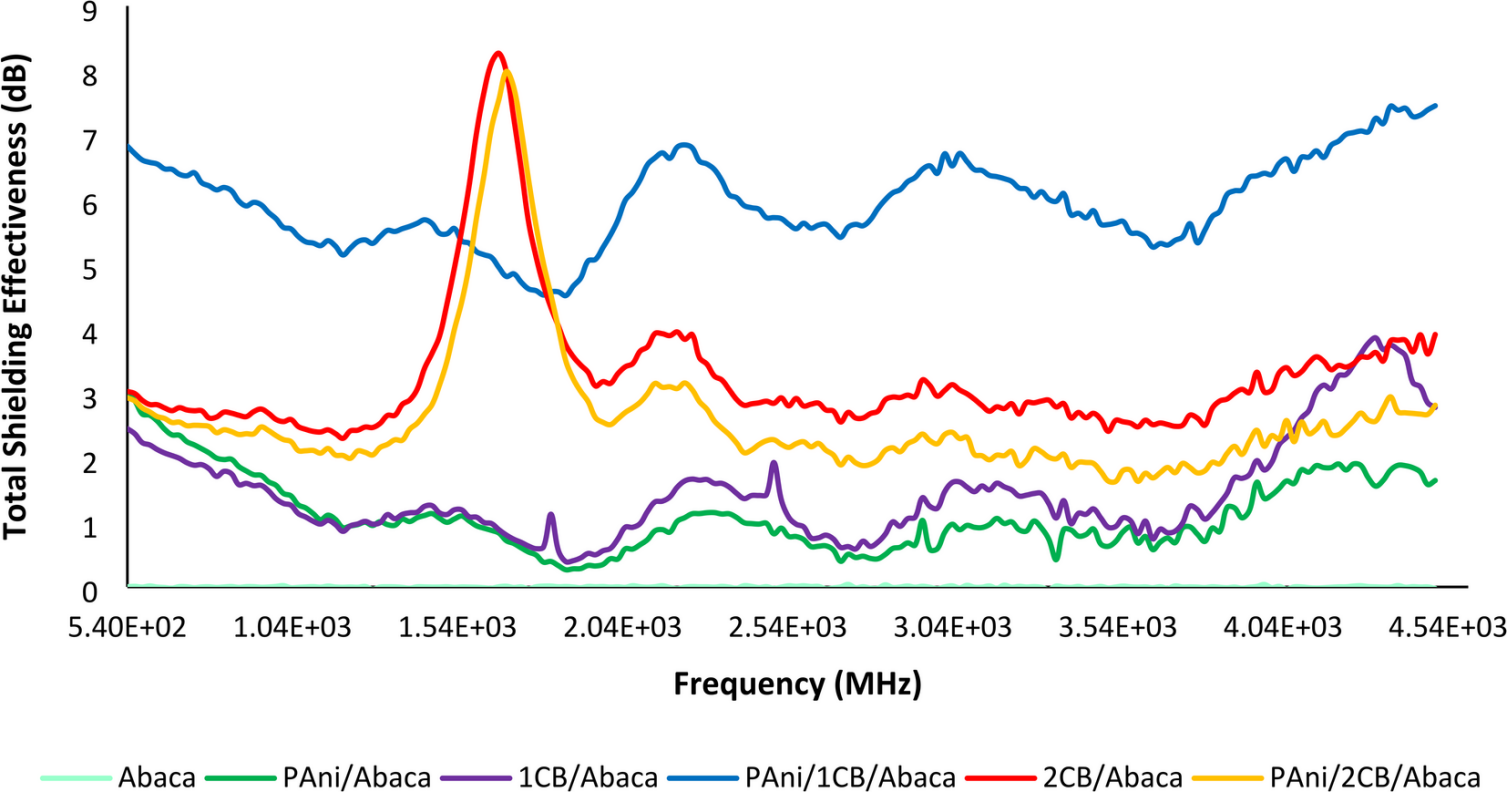
SE_T_ of the abaca composites.

The 2 g CB samples exhibit distinct frequency peaks, indicating selective responsiveness, while the 1 g CB sample lacks a prominent shielding effectiveness peak, likely due to variations in nanostructure distribution. The 2CB/Abaca and PAni/2CB/Abaca composites show similar peaks with attenuation values of 8.27 dB and 7.28 dB at 1.67 GHz and 1.69 GHz, respectively. The PAni/1CB/Abaca composite demonstrates stable EMI shielding effectiveness of 5.96 dB, with a 4.5 dB improvement attributed to enhanced PAni deposition on the Abaca surface by CB. This configuration offers consistent shielding across frequencies, with only ± 0.67 dB variation. According to EMI shielding theory, optimal conductive structure arrangement enhances internal electromagnetic wave scattering through electrical percolation^56^. The 2CB/Abaca composite’s performance is similar to the PAni/2CB/Abaca composite, likely due to challenges in PAni polymerization and limited attachment, which hindered effective percolation and conductive pathway formation. Figure 9 illustrates the Total Shielding Effectiveness SE_T_ of various Abaca fiber-based material combinations within the frequency range of 540 MHz to 4540 MHz. It can be observed that the PAni/CB/Abaca combination exhibits the highest SE_T_ value, approximately 8 dB at around 1.54 GHz, indicating excellent electromagnetic shielding (EMI shielding) performance. The 2CB/Abaca combination also shows a relatively high performance, around 8.5 dB at the same frequency. In contrast, reference materials such as pure Abaca and PAni/Abaca display significantly lower SE_T_ values, ranging from 1 to 2 dB, suggesting that the conductive filler materials such as CB (carbon black) and PAni (polyaniline) play a crucial role in enhancing the electromagnetic shielding effectiveness.

**Fig. 10:**
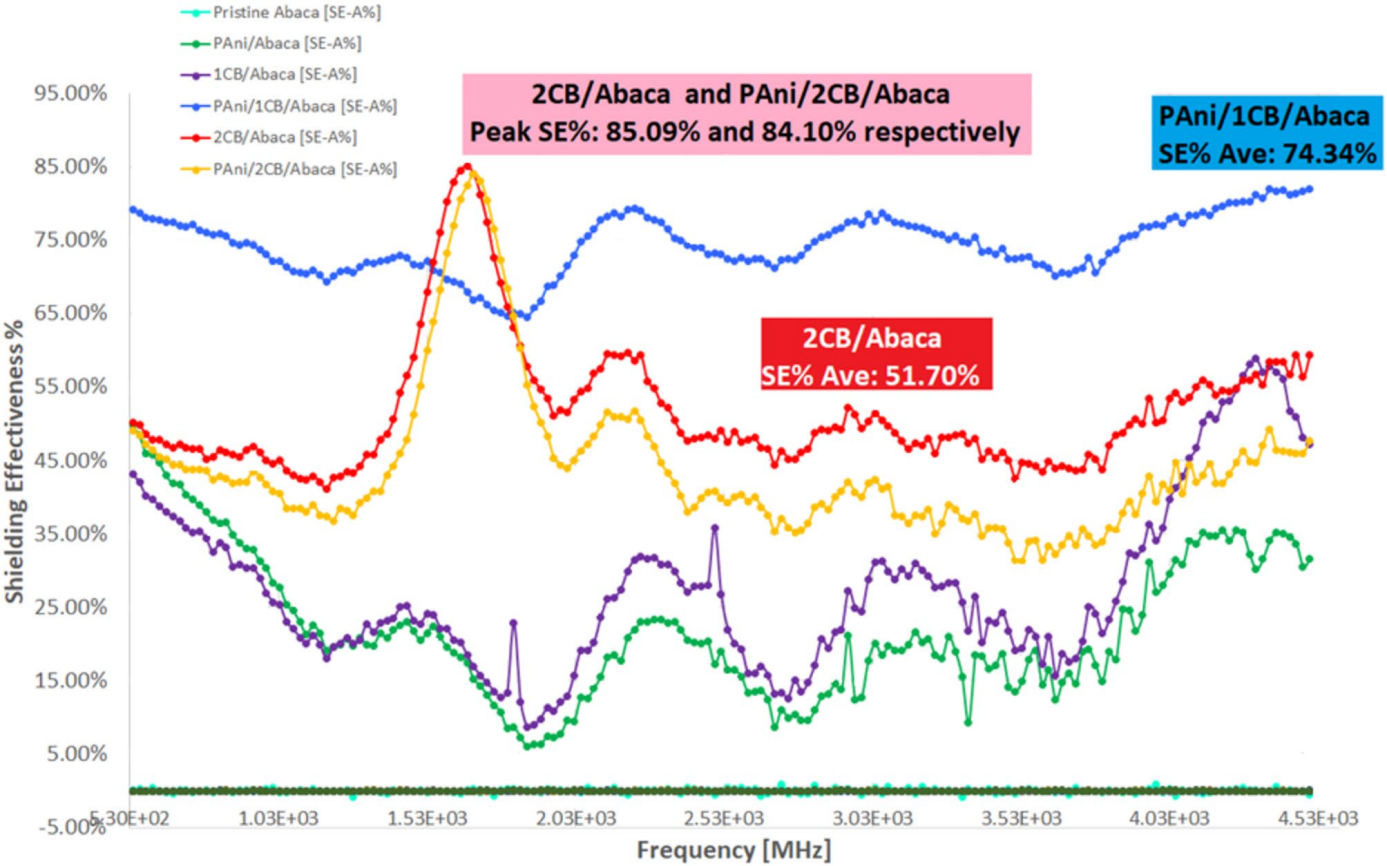
EMI SE% of the abaca composites.

Figure 10 shows the effectiveness of electromagnetic shielding in Abaca fiber-based composites varies depending on the combination of materials used, with the absorption mechanism as the dominant factor. PAni/1CB/Abaca showed the best performance with an average SE% of 74.34%, indicating a high ability to absorb UHF (Ultra High Frequency) radiation, while 2CB/Abaca and PAni/2CB/Abaca had an average SE% of 51.70% and 44.40%, with shielding peaks of 85.09% and 84.10% at certain frequencies, respectively. Composites based on one additional material, such as PAni/Abaca and 1CB/Abaca, have lower effectiveness with an average of 22.02% and 27.64%, indicating that the combination of PAni and CB provides a more optimal synergistic effect in increasing electromagnetic shielding. The basic structure of Abaca fibers that use taffeta webbing has gaps that can reduce shielding effectiveness, so increasing fiber density can potentially improve nanomaterial deposition and improve electromagnetic radiation absorption efficiency.

## Conclusions

The resistivities of the textile composites were assessed, revealing that PAni/1CB/Abaca exhibited the lowest resistivity (and thus the highest conductivity) at 891 Ω cm. 2CB/Abaca followed this at 1469 Ω cm, PAni/2CB/Abaca at 1503 Ω cm, PAni/Abaca at 2963 Ω cm, and finally, 1CB/Abaca with the highest resistivity of 5338 Ω cm. The Shielding Effectiveness (SE) analysis indicated that incorporating conductive nanomaterials enhanced the textiles, enabling effective electromagnetic shielding. The primary shielding mechanism across all composites was absorption rather than reflection. PAni/1CB/Abaca displayed the highest average SE at 5.96 dB, blocking 74.34% of EM radiation, followed by 2CB/Abaca (3.27 dB, 51.70%), PAni/2CB/Abaca (2.64 dB, 44.04%), 1CB/Abaca (1.47 dB, 27.64%), and PAni/Abaca (1.12 dB, 22.02%). The combination of PAni and CB notably enhanced SE compared to their individual counterparts. As the weight% increased, 2CB samples showed a peak in attenuation within the 1417 MHz to 1890 MHz range, with maximum attenuations of 8.27 dB (2CB/Abaca) and 7.98 dB (PAni/2CB/Abaca). The reason for this frequency-specific increase warrants further investigation, though literature suggests that layer formation within nanomaterial aggregates may enhance resonance and Multiple Internal Reflections in this frequency range.

## Data Availability

The data are available upon request to the corresponding author.
